# *In Vivo* Therapeutic Protection against Influenza A (H1N1) Oseltamivir-Sensitive and Resistant Viruses by the Iminosugar UV-4

**DOI:** 10.1371/journal.pone.0121662

**Published:** 2015-03-18

**Authors:** Eric J. Stavale, Hong Vu, Aruna Sampath, Urban Ramstedt, Kelly L. Warfield

**Affiliations:** 1 Integrated Biotherapeutics, Inc., Gaithersburg, Maryland, United States of America; 2 Unither Virology, LLC, Silver Spring, Maryland, United States of America; Boston University School of Medicine, UNITED STATES

## Abstract

Our lead iminosugar analog called UV-4 or *N*-(9-methoxynonyl)-1-deoxynojirimycin inhibits activity of endoplasmic reticulum (ER) α-glucosidases I and II and is a potent, host-targeted antiviral candidate. The mechanism of action for the antiviral activity of iminosugars is proposed to be inhibition of ER α-glucosidases leading to misfolding of critical viral glycoproteins. These misfolded glycoproteins would then be incorporated into defective virus particles or targeted for degradation resulting in a reduction of infectious progeny virions. UV-4, and its hydrochloride salt known as UV-4B, is highly potent against dengue virus *in vitro* and promotes complete survival in a lethal dengue virus mouse model. In the current studies, UV-4 was shown to be highly efficacious via oral gavage against both oseltamivir-sensitive and -resistant influenza A (H1N1) infections in mice even if treatment was initiated as late as 48-72 hours after infection. The minimal effective dose was found to be 80-100 mg/kg when administered orally thrice daily. UV-4 treatment did not affect the development of protective antibody responses after either influenza infection or vaccination. Therefore, UV-4 is a promising candidate for further development as a therapeutic intervention against influenza.

## Introduction

Pandemic, zoonotic and seasonal influenza viruses (INFV) remain a significant global threat to human health. Continuous evolution of INFV via both drift and shift in the viral genome results in generation of new strains each year and the potential for dangerous pandemics due to lack of immunity to these emerging, divergent viruses [1, 2]. While there are currently a few FDA-approved antiviral drugs that are available to the public, notably zanamivir and oseltamivir phosphate, these drugs as well as older drugs (amantadine and rimantadine) are known to rapidly generate drug-resistant variants, indicating that new antivirals for influenza are needed [3, 4].

There is currently great interest in novel, broad-spectrum antiviral strategies. Antiviral drugs targeting processes within the host that are required for viral replication, could offer a potential strategy for inhibiting different species within a viral family or even several different families of viruses. One such strategy, currently in development, is to target endoplasmic reticulum (ER) α-glucosidases I and II. These enzymes’ role in the host cell is to remove glucose residues from high-mannose *N*-linked glycans attached to glycoproteins, which allows for proper protein folding and transport within the cell [5]. Multiple enveloped viruses utilizing this cellular pathway to acquire their enveloped glycoproteins via ER budding have been shown to be sensitive to glucosidase inhibition [6]. Glucosidase inhibitors, such as castanospermine, inhibit glucose-trimming of *N*-linked oligosaccharides on glycoproteins, leaving them in a mono- or tri-glucosylated form [7]. Influenza viruses encode two glycoproteins, HA and NA, that are responsible for entry/fusion and exit of the virus particles from the cell, respectively [8–11]. For INFV, studies have shown that α-glucosidase inhibitors may play a role in the maturation of neuraminidase (NA) as a consequence of impaired binding to calnexin [12, 13], as well as recruitment of hemagglutinin (HA), which binds to calnexin in its monoglucosylated form [14, 15]. Despite consistent observations in altered glycosylation status of the critical NA and HA proteins, previous studies using iminosugars have been inconsistent in their demonstration of reduction in infectious INFV, which has been reported to be both virus strain and cell line specific [12, 16–19].

The iminosugar UV-4, a derivative of deoxynojirimycin (DNJ), has recently been shown to be a potent antiviral drug against dengue virus (DENV) *in vivo*. UV-4 has excellent bioavailability in BALB/c mice when delivered up to 200 mg/kg *per os* (PO) and protected 90% of interferon receptor-deficient AG129 mice from a lethal challenge in an antibody-dependent enhancement DENV model [20].

Here we describe the efficacy of UV-4 (administered as the free base or hydrochloride salt formulation, UV-4B) against lethal infection with a mouse-adapted oseltamivir-sensitive INFV A/Texas/36/91 (H1N1) and an oseltamivir-resistant strain of INFV A/Perth/261/2009 (H1N1). The therapeutic window (TW), minimum effective dose (MED), as well as the daily dosing regimen were determined, based on survival analysis. Mice were also tested for lung tissue-burdens and for memory antibody generation against INFV. UV-4 is both a safe and efficacious therapeutic candidate against INFV A (H1N1)-infected BALB/c mice.

## Materials and Methods

### Compounds

The active ingredient UV-4 was formulated for all studies in acidified water or in the form of the hydrochloride salt (*N*-9-methoxynonyl-deoxynojirimycin-HCl, aka UV-4B), which has a molecular weight approximately 11.4% larger than UV-4 base (ex.100 mg/ml of UV-4 formulated as 111.4 mg/ml of UV-4B). All drug concentrations henceforward are referred as the UV-4 base. UV-4 and oseltamivir phosphate (Roche) were reconstituted in sterile water for all *in vivo* studies.

### Viruses

Mouse-adapted influenza A/Texas/36/91 (H1N1) was obtained from the Baylor School of Medicine (kind gift of P. Wyde and B. Gilbert) and stocks prepared in house using infected-lung homogenates. Using 6–8 week old female BALB/c mice, the LD_90_ was determined to be 52 PFU/mouse. A Tamiflu-resistant strain of influenza A/Perth/261/2009 (H1N1) containing the H275Y mutation (source WHO Collaborating Centre for Reference and Research on Influenza Victorian Infectious Diseases Reference Laboratory (VIDRL)) was adapted to mice by serial passage in mouse lungs following intranasal (IN) administration of 100uL of virus. Using 6–8 week old female BALB/c mice, the LD_90_ was determined to be ~1.23x10^5^ PFU/mouse. The presence of the H275Y mutation and resistance to oseltamivir *in vitro* were confirmed by sequencing and using a TCID50 assay, respectively, in the mouse-adapted challenge stock.

### TCID assay for measuring infectious INFV titers in mice

Infectious influenza viruses from animal tissues were titrated in a tissue culture infectious disease assay (TCID_50_) using Madin-Darby canine kidney (MDCK) cells in replicates of nine. Lungs were pulverized in PBS (TissueRuptor, Qiagen), removed of debris via centrifugation, and titrated. Tissue culture-treated 96-well plates (Fisher Scientific) were seeded with Madin-Darby Canine Kidney cells (MDCK; ATCC) at 1x10^4^ cells per well in 100 ul of UltraMDCK (Lonza) supplemented with penicillin, streptomycin, L-glutamine, and 1 ug/ml of tosyl phenylalanyl chloromethyl ketone-treated trypsin (TPCK-trypsin; Sigma). Ten-fold dilutions of each lung or serum sample were incubated on the cells for 10 days before being fixed with 4% gluteric dialdehyde (Sigma) and stained with 1% crystal violet (Sigma) dissolved in 5% methanol. Cytopathic effect was scored visually and analyzed for TCID_50_ titer using a Spearman-Karber method [21]. All INFV titers were transformed from TCID_50_/ml to PFU/ml by multiplying TCID value by 0.69 [22]. The area under the curve (AUC) for viral titers in lungs over time was calculated with log-transformed data in GraphPad Prism using the Trapezoid Rule [23].

### Mice

BALB/c mice (average 20g starting weight, 6–8 weeks of age) of both sexes were used for a gross toxicity study. For INFV efficacy studies, 6–8 week-old female BALB/c mice (Charles River Labs) were microchipped for identification and temperatures (Bio Medic Data Systems) approximately 3 days prior to infection. Mice were infected with approximately 52 plaque-forming units (PFU) of A/Texas/36/91 (H1N1) diluted in phosphate-buffered saline (PBS) via IN administration. Mice were anesthetized (VetEquip IMPAC6) lightly using 2–5% v/v of isoflurane at ~2.5 L/min of O_2_. Mice were treated *per os* (PO) with UV-4 at various concentrations or oseltamivir phosphate at 10 ml/kg either two or three times daily (BID and TID, respectively). Weights, health scores and temperatures were monitored and recorded daily for the duration of the study on individual mice. A standard health scoring system from 1–7 was utilized where scores indicated the following: 1, healthy; 2, slightly ruffled; 3, ruffled; 4, sick; 5, very sick; 6, moribund; and 7, found dead. Mice were sacrificed at a health score greater than or equal to 5 or when a weight loss of >30% of their original weight was recorded. A total of 167 out of 750 mice used in the studies described here scored a 7. Animals were euthanized in accordance with the 2013 American Veterinary Medical Association (AVMA) Guidelines on Euthanasia using carbon dioxide exposure followed by cervical dislocation. All experimental procedures and studies were preapproved and performed according to guidelines set by the Noble Life Sciences Animal Care and Use Committee (IACUC). Survival data was analyzed in GraphPad Prism using log-rank analysis.

### Antibody responses to influenza following UV-4 treatment

In the first study, mice were infected with influenza (INFV) A/Texas/36/91 (H1N1) to determine whether there is an effect on development of antibody responses in UV-4-treated mice. Groups of 6–8 week old female BALB/c mice received UV-4 (n = 10) or vehicle (n = 20) TID for 10 days starting 1 hour prior to a challenge with 1xLD_90_ of INFV. Serum samples were collected via tail vein nick on days-3, 15, 30, and 120 relative to INFV challenge and hemagglutination inhibition (HAI) serum titers were determined in all the samples.

In a second study, 20 BALB/c mice were vaccinated intramuscularly with 50 μL of the Fluvirin 2010/2011 influenza vaccine containing the strains A/CA/07/2009, A/Perth/16/2009, and B/Brisbane/60/2008 on days 0, 14, and 28, while 20 control mice received only 50 μL Dulbecco’s phosphate-buffer saline (PBS) intramuscularly. Oral UV-4 treatment was administered at 100 mg/kg TID in half (n = 10) of each vaccinated and vehicle-only control groups of mice while the vehicle was given to the other half of each group on the first day of vaccination for a total of 10 days (30 doses on study days 0–10). Sample collection was conducted on days 7, 14, and 28, and the animals were exsanguinated on day 42. To determine immune responses to influenza after infection or vaccination, a standard hemagglutination inhibition assay (HAI) was utilized. Mouse serum samples were treated with Receptor Destroying Enzyme (RDE) overnight at 37°C to remove any non-specific agglutination in the sera that could interfere with proper titer determination. After heat-inactivation of the RDE for 1 hour at 56°C, RDE-treated mouse serum samples were serially diluted by a factor of two in DPBS. Serum samples were pre-incubated with antigen (INFV/A/Texas/36/91 antigen from NIBSC or Fluvirin 2010/2011 TIV influenza vaccine) for one hour at room temperature before adding 0.5% chicken red blood cells to the mixture. HAI was visualized one hour after the final incubation; wells positive for HAI were indicated by observation of a lattice structure. Statistical analysis of the HAI titers in the infection study could not be performed due to the small number of surviving animals in the vehicle control group (n = 2); a 2-way repeated-measures (days of bleed) ANOVA was used to test for difference in the HAI titer between the two groups in the vaccine study (GraphPad).

## Results

### Gross safety and toxicity of UV-4B in BALB/c mice

**Fig 1:**
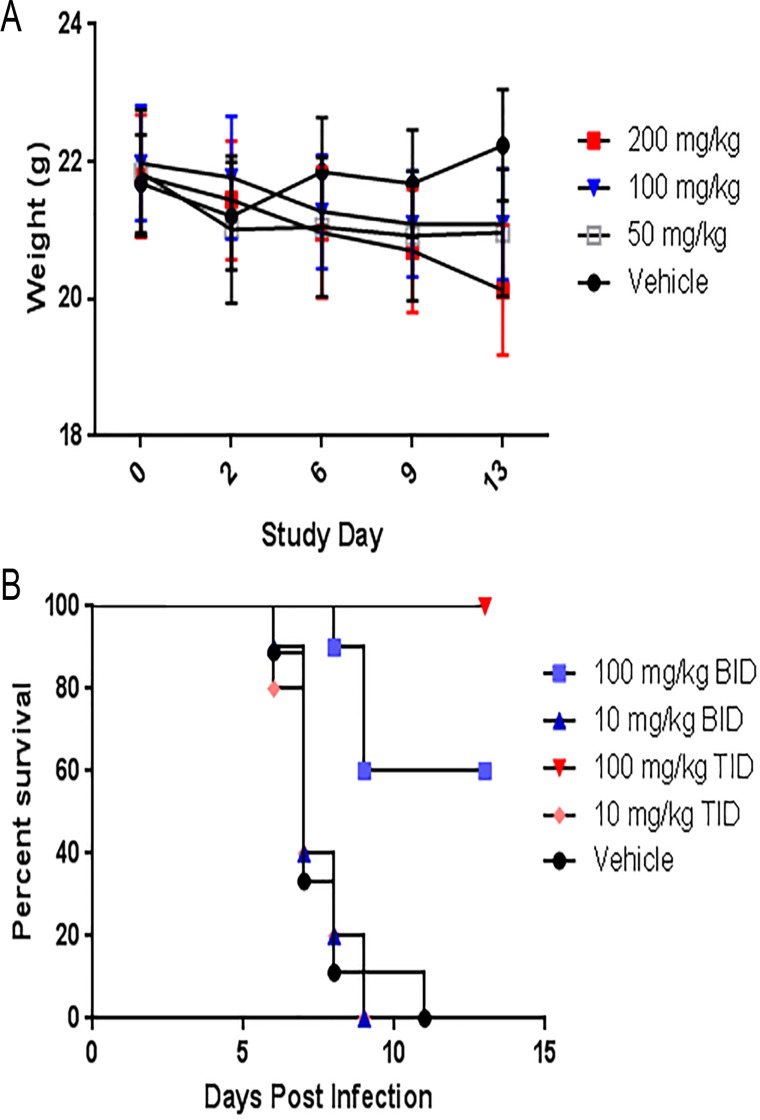
Protective efficacy of UV-4 dosing TID vs BID against influenza-infected mice. **(A)** To examine the effect of UV-4 administration on the weight of healthy BALB/c mice (5 males and 5 females per group), UV-4 was given TID via oral gavage at 0, 50, 100 or 200 mg/kg for 14 days (study days 0–13). The mean weights with standard deviation for each group are plotted on the study days that they were measured. **(B)** Groups of mice (n = 10) received the first treatment dose of compound in water 1 h before an INFV infection with mouse-adapted influenza A/Texas/36/91 at a dose of ~1LD_90_. Graph shows treatment with 100 or 10 mg/kg of UV-4 two or three times a day for 10 days, PO, at 12 or 8 hour increments or vehicle control given thrice daily for 10 days. Survival data is plotted as percent survival against days post infection.

Weight loss is a major criteria for early endpoints in influenza models. To examine the effect of UV-4 administration on the weight of healthy BALB/c mice, UV-4 was given TID via oral gavage at 0, 50, 100 or 200 mg/kg for 14 days (study days 0–13). Minor (4 to 7.6%) but statistically significant weight loss was observed in mice dosed at all levels of UV-4 compared to animals given vehicle only (Fig. 1A) although animals followed for an additional treatment-free 10 days recovered their body weights (not shown). This weight loss correlated with decreased food consumption during the treatment period but may also be related to intestinal distress, as has been noted for other iminosugar treatments such as miglustat [24].

### Antiviral activity of UV-4 *in vivo* against INFV

While partial protection against dengue challenge was shown with BID dosing, more robust efficacy was observed with TID dosing [20]. To test for efficacy of UV-4 against influenza, mice were treated PO with 10 or 100 mg/kg of UV-4 beginning one hour prior to a lethal challenge with 1 LD_90_ (~52 PFU) of INFV A/Texas/36/91 (H1N1). Mice continued to be dosed with UV-4 BID or TID for 7 days and observed for up to 14 days for morbidity and mortality. The group of infected mice dosed BID with 100 mg/kg exhibited 60% survival, while the group treated with 100 mg/kg TID exhibited 100% survival (Fig. 1B). Survival in the groups dosed with 10 mg/kg was not statistically different from the vehicle control (mean time to death or MTD = 7 days for each group and have 0% survival). Therefore, UV-4 was most efficacious against a 1xLD_90_ challenge of INFV A/Texas/36/91 when delivered at 100 mg/kg TID (p = 0.04 comparing survival in 100 mg/kg administered TID and BID).

### Minimal effective dose and therapeutic window of efficacy of UV-4 in INFV-infected mice

**Fig 2:**
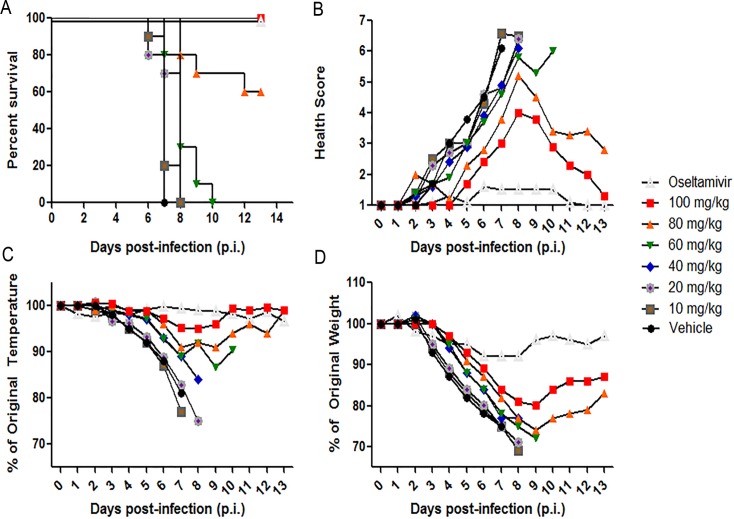
Determination of minimal effective dose of UV-4 against influenza. Female BALB/c mice were infected with ~1xLD_90_ of INFV A/Texas/36/91 (H1N1) via IN instillation. Mice (n = 10/group) were orally treated TID for 10 days with 100, 80, 60, 40, 20 or 10 mg/kg of UV-4 or vehicle or twice daily for 5 days with oseltamivir phosphate at 20 mg/kg. (**A)** Survival data is plotted as percent survival against days post infection. **B)** The mean health score for each group are plotted against days post infection. **C)** The mean percent temperatures for each group are plotted compared to their percent temperature on day 0 (baseline) against days post infection and **D)** The mean percent weights for each group are plotted compared to their percent weight on day 0 (baseline) against days post infection.

To determine the minimum effective dose (MED) of UV-4 against influenza, mice were challenged with ~1XLD_90_ of mouse-adapted INFV A/Texas/36/91 (H1N1) and administered 10, 20, 40, 60, 80, or 100 mg/kg of UV-4 TID starting one hour before infection. The study included a positive control group that was administered orally BID with 20 mg/kg of oseltamivir phosphate (Tamiflu). Survival (Fig. 2A), general health (Fig. 2B), body temperatures (Fig. 2C), and weights (Fig. 2D) were monitored daily for 14 days total (study days 0–13). All vehicle control animals succumbed to the infection with a mean survival of 7 days. Groups dosed with 100 mg/kg of UV-4 or 20 mg/kg of oseltamivir phosphate showed 100% survival at day 14, while the group dosed with 80 mg/kg of UV-4 displayed 60% survival. Groups which were treated with lower doses of UV-4 displayed 0% survival and a mean survival time between 7–8 days. All groups dosed orally, TID, with UV-4 with doses of 100, 80, 60, 40, or 10 mg/kg showed significant survival rates (p<0.0001 for groups treated with oseltamivir phosphate, 100, or 80 mg/kg and p<0.005 for groups treated with 60 or 40 mg/kg based on percent survival and survival time). The MED (defined as providing 100% survival) using the mouse-adapted INFV A/Texas/36/91 (H1N1) was found to be 100 mg/kg of UV-4 TID. Mice treated with UV-4 had higher health scores and more weight loss than those treated with oseltamivir phosphate but were nonetheless protected against lethal challenge at the MED.

To determine the therapeutic window of treatment with UV-4, groups of mice were treated orally TID with UV-4 at 100 mg/kg starting at-1, 24, 48, 72, 96, or 120 hours relative to infection. Treatment regimens of 7 or 10 days were examined. Positive control groups of mice were treated with 20 mg/kg of oseltamivir phosphate twice daily for 5 days via oral gavage starting at-1, 24, 48, 72, 96, or 120h relative to infection. One group of 10 mice served as a vehicle control and was dosed orally with water TID for 10 days starting at-1h relative to infection. A summary of survival results (a combination of results from two studies having 10 mice per group per study) are presented in Table 1. Treatment with UV-4 provided significant protection (p<0.05) against INFV infection as compared to vehicle control treated animals, when given for 7 or 10 days, with treatment starting as late as 72–96 hours after infection (Table 1). When compared to dosing with water alone, all groups treated with oseltamivir starting as late as 120 hours after infection showed a significant benefit in survival (p<0.05, Table 1).

**Table 1:** Survival of mice treated with UV-4 for 7 or 10 days starting at varied times relative to challenge.

Initiation of Treatment	Vehicle, 10 days	UV-4, 7 Days		UV-4, 10 Days		Oseltamivir, 5 days	
	% Survival	% Survival	P value	% Survival	P value	% Survival	P value
-1h	20%	-	-	-	-	-	-
-1h	-	90%	>0.0001	100%	>0.0001	100%	>0.0001
+24h	-	85%	>0.0001	95%	>0.0001	67%	0.0005
+48h	-	50%	0.0231	55%	>0.0001	55%	0.0022
+72h	-	40%	0.0423	40%	0.0078	70%	0.001
+96h	-	45%	0.0494	10%	0.08	40%	0.001
+120h	-	30%	0.25	30%	0.39	10%	0.013

Groups of BALB/c mice (n = 20, combined data from 2 experiments each having 10 animals/group) received the first treatment dose of 100 mg/kg of UV-4 or 20 mg/kg of oseltamivir phosphate at-1, 24, 48, 72, 96, or 120 h or vehicle control at-1h relative to infection with ~1 LD_90_ of INFV A/Texas/36/91 (H1N1) via IN instillation. Treatment began at the time point indicated relative to challenge with UV-4 dosing continued TID every 8 hours for a total of 7 or 10 days, while oseltamivir phosphate treatment continued BID for 5 days. Percent survival is presented along with statistical comparison using log rank tests of each treated group compared to the vehicle only control group.

### Viral loads are decreased in infected mice treated with UV-4

**Fig 3:**
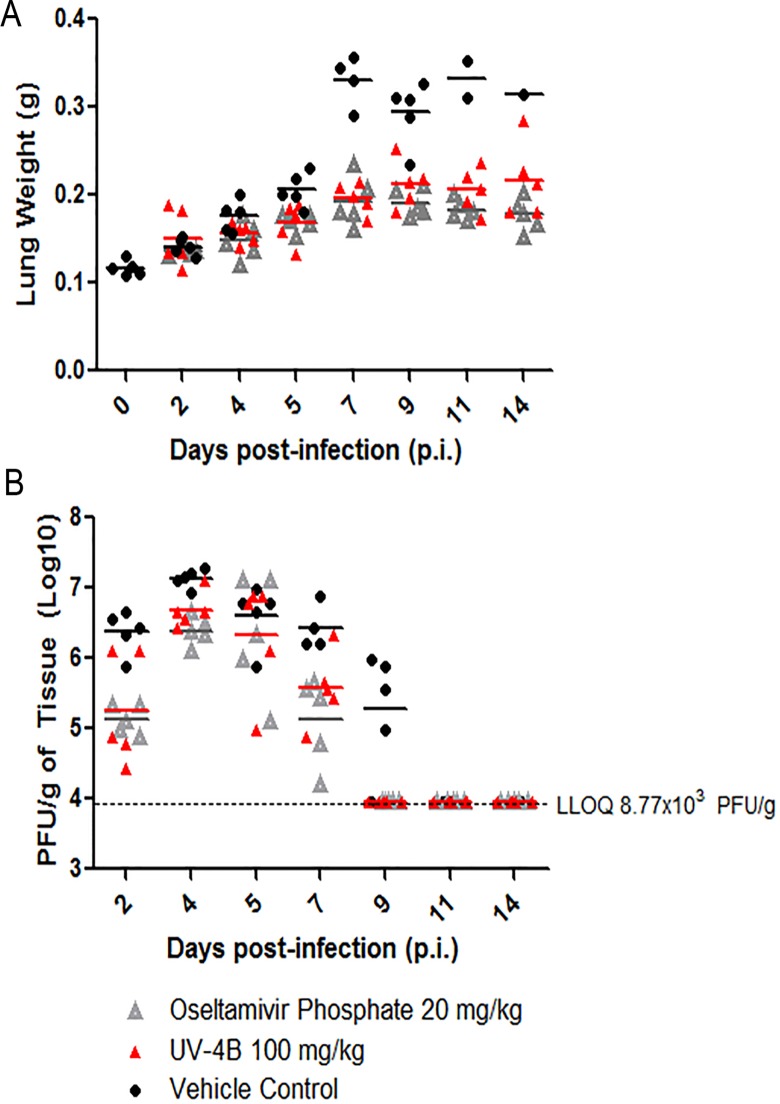
Analysis of viral titers in UV-4 treated mice infected with INFV A/Texas/36/91 (H1N1). Lung and serum from mice treated with 100 mg/kg of UV-4, oseltamivir or vehicle-alone were collected on 2, 4, 5, 7, 9, 11, or 14 days post infection and analyzed for viral load using a TCID_50_ assay. **(A)** Wet lungs were weighed in grams and plotted individually. **(B)** Viral titers were log-transformed in GraphPad Prism and mice were plotted individually.

To examine the effect of UV-4 treatment on virus replication, groups of mice were treated orally TID with vehicle or UV-4 at 100 mg/kg for 10 days or 20 mg/kg of oseltamivir phosphate PO BID for 5 days, starting at-1 hour relative to infection. Lungs and serum were harvested from five mice per group (unless no mice remained) on days 2, 4, 5, 7, 9, 11, or 14 post-infection. As a negative control, lungs and serum from one group of 5 naive mice was harvested of on day 0. The weights of lung samples collected from each mouse are shown in Fig. 3A. Lungs and serum from each harvested group were titrated in a TCID_50_ assay on MDCK cells to quantify viral loads in relative tissues (Fig. 3B and data not shown). On day 2 post-infection, the vehicle-treated mice had ~ 1 log higher viral lung titers per gram (6.37 log10) than UV-4- and oseltamivir-treated mice (5.26 and 5.12 log10, respectively). This pattern was also observed on days 4 and 7, post infection, with UV-4- and oseltamivir-treated mice having significantly lower mean titers per gram of tissue than vehicle-treated mice. Mean titers for all three treated groups appear similar on day 5 post-infection with 6.32, 6.32, and 6.61 (log10) PFU per gram of tissue for UV-4, oseltamivir-, and vehicle-treated mice, respectively. The area under the curve (AUC) for viral lung titers was determined to be 10.89 units for the vehicle-treated mice compared to 7.4 and 6.6 units for UV-4 and oseltamivir-treated groups, respectively. As expected, no virus was detected in any of the serum samples in any mice.

### UV-4 protects against challenge with a lethal tamiflu-resistant INFV

**Fig 4:**
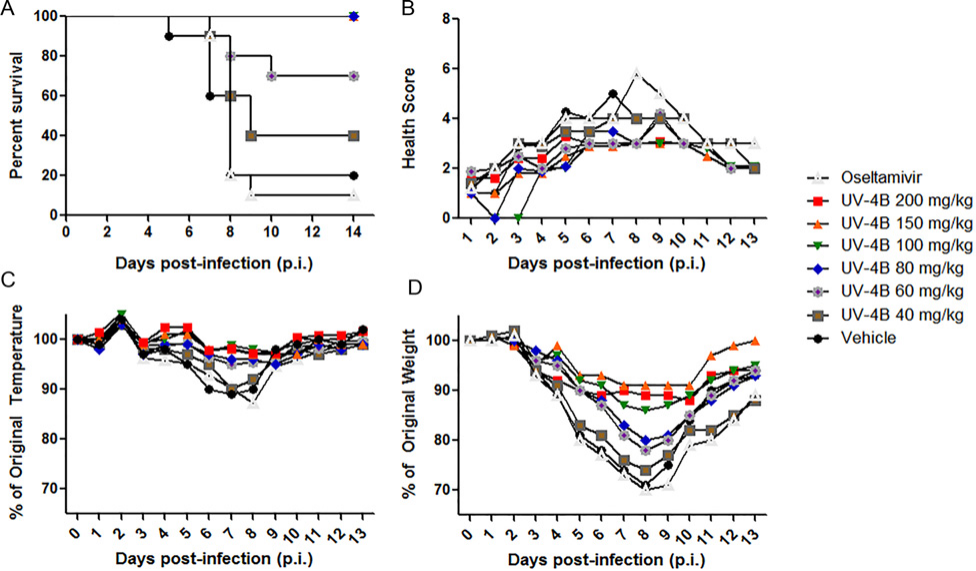
Determination of minimal effective dose of UV-4 against mouse-adapted Tamiflu-resistant strain of influenza A/Perth/261/2009 (H275Y). Female BALB/c mice were infected with ~1xLD_90_ of mouse-adapted Tamiflu-resistant strain of influenza A/Perth/261/2009 (H275Y) (H1N1) via IN instillation. Mice (n = 10/group) were orally treated TID for 10 days with 200, 150, 100, 80, 60, or 40 mg/kg of UV-4 or vehicle only or twice daily for 5 days with oseltamivir phosphate at 20 mg/kg. (**A)** Survival data is plotted as percent survival against days post-infection. **B)** The mean health score for each group are plotted against days post infection. **C)** The mean percent temperatures for each group are plotted compared to their percent temperature on day 0 (baseline) against days post infection. **D)** The mean percent weights for each group are plotted compared to their percent weight on day 0 (baseline) against days post infection.

We determined the MED of UV-4 in mice during lethal INFV infection (~1xLD_90_ of a Tamiflu-resistant strain of influenza A/Perth/261/2009 (H275Y) administered intranasally) by administering 40, 60, 80, 100, 150 or 200 mg/kg of UV-4 TID for 10 days starting one hour before infection. A negative control group received water treatment only (vehicle) and the last group received oseltamivir phosphate (Tamiflu) at a dose of 20 mg/kg BID for 5 days. Survival (Fig. 4A), general health (Fig. 4B), body temperatures (Fig. 4C), and weights (Fig. 4D) were monitored daily for 14 days total (study days 0–13). Complete survival was displayed in groups treated with 200, 150, 100, and 80 mg/kg of UV-4. In the groups treated with 60 and 40 mg/kg, 70 and 40% survival was observed, respectively. The control groups, treated with oseltamivir phosphate or water, displayed 10 and 20% survival, respectively. The MED against a mouse-adapted Tamiflu-resistant strain of INFV virus A/Perth/261/2009 (H275Y mutant) was determined to be 80 mg/kg.

### Generation of protective immune responses to INFV after UV-4 treatment

**Fig 5:**
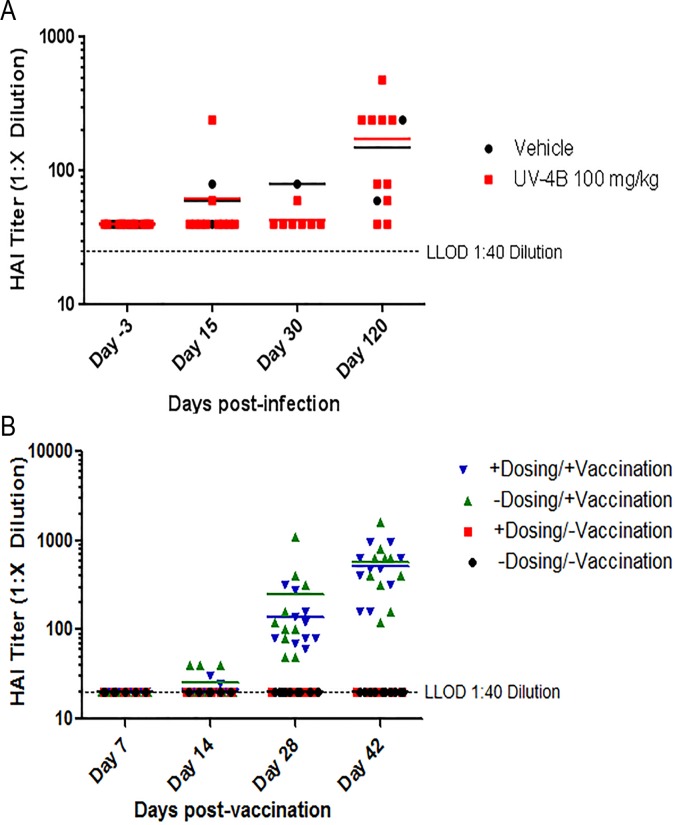
Influence of UV-4 on antibody titers induced by influenza infection or vaccination. (A) Groups of mice received the first treatment dose of compound (n = 10) or water (n = 20) starting 1 hour before an IN infection with INFV. Serum samples were collected on days-3, 15, 30, and 120 post-infection. Samples were treated with receptor-destroying enzyme II before being added to chicken red blood cells for determination of HAI activity. (B) Mice were split into 4 groups of 10 mice either vaccinated with or without UV-4 treatment or no vaccination with or without UV-4 treatment; where relevant, mice were dosed orally with 100 mg/kg of UV-4, TID, for 10 days total. Mice receiving vaccine were injected on days 0, 14, and 28 with 50 μL of Fluvirin 2010/2011 (Novartis, containing A/California/07/2009, A/Perth/16/2009, and B/Brisbane/60/2008) vaccine intramuscularly. Mice were bled on days 0, 14, 28 and 42 and serum of mice was tested for inhibition of hemagglutination against homologous seasonal flu vaccine Fluvirin 2010–2011.

Following infection with INFV, protective immunity is generated to the homologous virus strain and can be assessed by development of antibodies that inhibit hemagglutination activity *in vitro* (level of ≥1:40 is considered protective). To determine whether UV-4 treatment altered the development of protective immune responses to INFV, as measure by HAI, two studies were performed. In the first study, mice were infected with ~1xLD_90_ of influenza A/Texas/36/91 (H1N1) 1 hour after their first dose of UV-4. All of the mice (n = 10) treated with UV-4 survived through day 13 post-infection while 10% of the control mice (two of 20) survived. Serum samples were collected on days-3, 15, 30, and 120 post-infection. Samples were treated with receptor-destroying enzyme II before being added to chicken red blood cells for determination of HAI activity (Fig. 5A). Average HAI titers for the UV-4 treated animals were 62, 43 and 174 on days 15, 30 and 120, while the vehicle control treated animals had similar average HAI titers of 60, 80 and 150 on the same days. The small number of surviving animals in the vehicle control group did not allow for statistical comparison between the two treatment groups.

In the second study, UV-4 was given to mice vaccinated with a commercial INFV vaccine to determine the impact on development of vaccine-induced anti-INFV antibodies. The compound was given by the oral route TID for a total of 10 days after intramuscular vaccination with the 2010/2011 Fluvirin influenza vaccine. The antibody titers in the mouse sera were evaluated using a HAI test. No significant difference was observed when testing serum samples for HAI on days 0, 14, 30, and 42 between groups that were vaccinated with Fluvirin and were either treated or untreated with UV-4 (Fig. 5B). A 2-way repeated-measures (days of bleed) ANOVA tested between two vaccinated groups (GraphPad) revealed p = 0.77 for interaction indicating no statistical significance between UV-4-treated and untreated vaccinated groups. The study indicated that UV-4 treatment did not have an impact on the immune response to the seasonal flu vaccine Fluvirin 2010–2011 TIV.

## Discussion

We have shown that our lead candidate iminosugar UV-4 is protective against lethal INFV A (H1N1) disease in mice. Dosing with UV-4 thrice daily resulted in better levels of protection than twice daily dosing. We have previously determined the pharmacokinetic parameters of UV-4 in mice and found that the T_max_ (time to maximum concentration) in plasma was 15 minutes with a C_max_ (maximum concentration) of 91 ng/mL, a T_1/2_ (terminal elimination half time) of 5.14 hours, AUC_inf_ (area under the curve (t = 0 to infinite)) of 129,017 hr*ng/mL and 84.2% bioavailability [20]. The finding that more frequent dosing is required for more robust protection is in line with the short half-life of UV-4 and our previous studies demonstrating higher efficacy against dengue by UV-4 when given TID. UV-4 was effective when given up to 72–96 hours after infection with a minimum effective dose of 80–100 mg/kg TID. UV-4 significantly reduced viral titers in the lungs of influenza-infected mice with a similar AUC to an approved drug oseltamivir, albeit at a higher dose level. The higher dose level of UV-4 required for efficacy as compared to that of oseltamivir likely reflects their divergent targets. Oseltamivir functions as an antiviral for INFV through direct, competitive inhibition of a single viral-encoded neuraminidase compared with UV-4 that requires saturation of three mammalian glucosidases widely distributed among influenza target and non-target cells. These two drugs have different structures, adsorption, distribution, metabolism and excretion profiles, and mechanisms of action and, thus, it is not unexpected that they require different doses and schedules to alter the outcome of influenza infection.

While previous studies with multiple glucosidase inhibitors have clearly shown effects on the glycosylation of INFV glycoproteins, the demonstration of inhibition of viral replication has been more variable and strain- and cell type-dependent. Castanospermine treatment of MDCK cells reduced viral titers of a reassortant NWS-N8 (H1N1 virus replaced with NA gene from N8 of A/Duck/Ukraine/1/63) [12] but not H1N1 viruses including the wild-type INFV A/NWS/33 strain or INFV A/PR/8/34 [16, 17]. Bromoconduritol reduced virus replication of an avian INFV A/Rostock (H7N1) but not INFV A/PR/8/34 (H1N1) in chick embryo cells. INFV A/Rostock (H7N1) was not inhibited by *N-*methyl-deoxynojirimycin in the same cells [18, 19]. Additionally, recent studies have suggested a strain-dependent inhibition of INFV replication using iminosugars *N-*butyl-deoxynojirimycin and *N-*nonyl-deoxynojirimycin, which are related to UV-4 [25]. Of note, there is no evidence in the literature for *in vivo* antiviral activity of iminosugars against INFV A (H1N1); however, we have clearly shown evidence for antiviral activity of UV-4 against two H1N1 isolates *in vivo*. These findings suggest that one should proceed cautiously when trying to extrapolate from results of *in vitro* studies of antiviral activity by iminosugars to *in vivo* activity profiles. A future direction of our work will be to examine the activity of UV-4B against a broader panel of influenza viruses including INFV A H3N2, H5N1 and H7N9 isolates as well as INFV B both *in vitro* and *in vivo*.

Influenza viruses encode two glycoproteins, HA and NA, that are critical for multiple steps in the viral life cycle [8]. HA, a lectin, mediates vial attachment of the viral particle via sialic acid receptors on the target cell surface and, once inside the cell, is responsible for fusion of the viral and endosomal membrane [9]. NA has enzymatic activity whereby it removes sialic acid from the cell surface and allows efficient release of the progeny virus from infected cells [10, 11]. Glucosidase inhibitors have been reported to inhibit interactions of HA and NA with calnexin and calreticulin [13, 14, 16, 26], ER-resident proteins that mediate proper co-translational folding of both viral glycoproteins [13, 27–29]. Related iminosugars NB-DNJ and NN-DNJ, which both have varied degrees of activity against INFV in vitro, do not affect the amount of HA and NA proteins synthesized but do cause an increase in the amount of tri-glucosylated glycoproteins in virus particles. While treatment of INFV infected cells with the more potent NN-DNJ caused a reduction in the sialidase activity, by using virus assortants, the activity appeared to be dependent on the HA [25]. The exact target of UV-4 in the viral life cycle has not yet been determined but will be the subject of future mechanism of action studies.

Due to the mechanism of action of iminosugars, one concern for using them as a treatment for viral diseases is the impact on generation of protective immunity. UV-4 presumably affects one or more of the influenza glycoproteins by altering their glycosylation; therefore, it is possible that treatment with UV-4 could have an effect on the protein folding and conformation of protective antigens such as HA [25]. Treatment with iminosugars could also theoretically have an impact on production of glycosylated host proteins such as immunoglobulins. For these reasons, it was important to examine immunity to the homologous strain after infection in the context of UV-4 treatment. In the context of a live virus infection, mice treated with UV-4 were able to mount protective antibody responses measured by HAI with similar kinetics and magnitude as those treated with water alone although the number of survivors in the control group were small (n = 2) due to the viral challenge dose of 1xLD_90_. As the LD_90_ of the mouse-adapted INFV A/Texas/36/91 strain is ~50 PFU, lowering the challenge dose further would likely result in not all mice being infected and, thus, uninterpretable results. Therefore, we performed a second experiment using influenza vaccination in the context of UV-4 treatment as a confirmation of the initial study using live infection. These findings are similar to those observed after UV-4 treatment of dengue infected mice where development of IgM and IgG responses were not different to vehicle treated mice [20].

A recent study of siblings diagnosed with the rare congenital disorder of glycosylation type IIb (CDG-IIb) provides further evidence that iminosugars, such as UV-4B, would be effective against viral infections [30]. CDG-IIb is a genetic disorder affecting the *N*-glycosylation process and, specifically, results in a defect in the processing of *N*-glycans due to an absence of functional α-glucosidase I. Cells from the siblings with CDG-IIb and healthy donors were collected and tested for susceptibility to four strains of HIV, Influenza A (H1N1), and Adenovirus type 5. In support of our findings, influenza A (H1N1) was unable to productively infect cells derived from the two siblings unlike those from healthy donors. Such findings were consistently observed for HIV and adenovirus. Extrapolating these findings to iminosugars suggests that compounds that effectively interfere with glycosylation via inhibition of α-glucosidases should be effective in suppressing viral replication in those infected with viruses that depend on glycosylation to enter or exit host cells.

The iminosugar class of molecules has long held the possibility of antiviral activity against a diverse set of enveloped, glycosylated viruses [6, 31] and significant data has been generated in animal models of flavivirus infection such as dengue and Japanese encephalitis virus [reviewed in [6]]. The *in vivo* antiviral activity of α-glucosidase inhibitors against INFV had not been previously demonstrated. We have now demonstrated that the iminosugar UV-4 has antiviral activity *in vivo* against both INFV and dengue [20], indicating that UV-4 is an inhibitor of multiple virus families. More work needs to be done to show whether it has even broader antiviral activities such as the well described glucosidase inhibitor castanospermine [32–35]. Several iminosugars have now been tested safely in the clinic for antiviral activity in humans against viruses such as human immunodeficiency virus [36], hepatitis C virus [32] and dengue [37] with only modest reductions in viral titers observed. Next generation iminosugar molecules such as UV-4 with greater potency should be pursued as clinical candidates with promise as antiviral agents that are effective against viruses that exploit host glycosylation pathways and have a reduced likelihood for generation of antiviral resistance due to targeting of host-based mechanisms.
